# Roles of METTL3 and NLRP3 in pyroptosis and prospects in SCIRI

**DOI:** 10.3389/fimmu.2025.1552704

**Published:** 2025-04-01

**Authors:** Xiaoqing Guan, Fengyi Zhang, Ning Zhang, Guangchun Li, Fei Yin

**Affiliations:** ^1^ Departments of Orthopedics, China-Japan Union Hospital of Jilin University, Changchun, China; ^2^ Departments of Orthopedics, Jilin Province People’s Hospital, Changchun, China

**Keywords:** SCIRI, pyroptosis, NLRP3, METTL3, SCI

## Abstract

Spinal cord ischemia-reperfusion injury (SCIRI) leads to severe neurological deficits, with pyroptosis emerging as a key driver of inflammation and neuronal death. Recent studies suggest that methyltransferase-like 3 (METTL3), a critical RNA methyltransferase, may regulate the nucleotide-binding oligomerized structural domain-like receptor protein 3 (NLRP3) inflammasome activation via N6-Methyladenosine (m6A) modification, yet direct evidence in SCIRI remains limited. This review synthesizes current knowledge on the METTL3-NLRP3 axis in pyroptosis, explores its therapeutic potential, and identifies critical research gaps for future investigation.

## 1 Introduction

Spinal cord ischemia-reperfusion injury (SCIRI) is a serious complication after aortic surgery or traumatic spinal cord injury (SCI) that leads to irreversible neurological deficits and has a profound impact on the quality of life of patients (1). The pathophysiology of SCIRI involves a cascade of events triggered by reperfusion, including oxidative stress, inflammatory activation, and programmed cell death (2). Among these, pyroptosis, a programmed cell death in the form of lysis driven by inflammatory vesicles, is considered a key factor in neuronal loss and secondary damage in SCIRI (3). Unlike apoptosis and necrosis, pyroptosis is characterized by pore formation mediated by gasdermin family proteins, rapid plasma membrane rupture, and the release of pro-inflammatory cytokines such as interleukin-1β (IL-1β) and interleukin-18 (IL-18), which exacerbate neuroinflammation and tissue damage (4). This unique interplay between cell death and inflammation makes pyroptosis a potential therapeutic target in SCIRI.

Central to pyroptosis is the nucleotide-binding oligomerized structural domain-like receptor protein 3 (NLRP3) inflammasome, a multiprotein complex that senses cellular stress and initiates caspase-1-dependent inflammatory signaling pathways (5). In SCIRI, NLRP3 activation is strongly correlated with disease severity, as evidenced by elevated NLRP3 levels in rodent models (6). However, the upstream regulators of NLRP3 in SCIRI remain unclear, which limits the development of targeted therapies. In recent years, breakthroughs in epitranscriptomics have highlighted the role of RNA modifications in inflammatory diseases. Methyltransferase-like 3 (METTL3) is an important RNA modification enzyme, which is mainly responsible for adding N6-methyladenosine (m6A) to RNA molecules to regulate mRNA stability, splicing and translation, thereby affecting a variety of pathological processes (7). Interestingly, METTL3 is associated with the activation of the NLRP3 inflammasome. However, whether this METTL3-NLRP3 axis plays a role in SCIRI and how it interacts with pyroptosis in nerve cells remains to be explored.

This review synthesizes emerging evidence on the METTL3-NLRP3 interaction in pyroptosis and proposes its relevance to SCIRI. We first elaborate on the mechanism of NLRP3 inflammasome activation, highlighting its unique role compared to other cell death pathways. Next, we discuss the regulatory role of METTL3 on NLRP3, focusing on M6A-dependent mRNA regulation and interactions with inflammatory signaling. Finally, we evaluated therapeutic strategies targeting the METTL3-NLRP3 axis to translate insights from mechanistic studies into therapeutic approaches with potential for clinical application. This study aims to advance the research on the epitranscriptomic regulation of neuroinflammatory RNA and promote the precision treatment of SCIRI.

## 2 NLRP3 inflammasome in SCIRI

### 2.1 Composition and activation mechanism of the NLRP3 inflammasome

**Figure 1:**
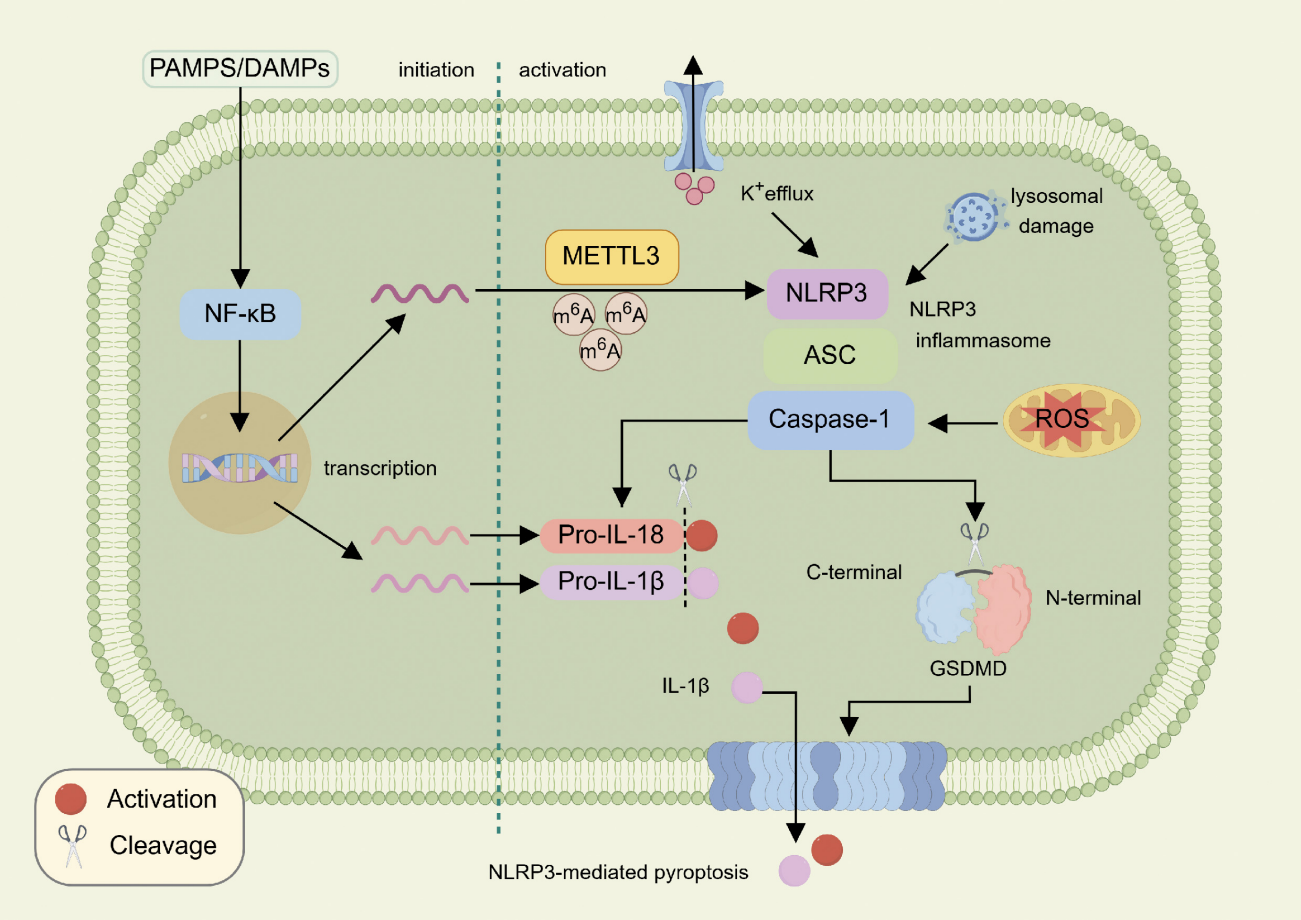
Interaction of METTL3 and NLRP3 in pyroptosis.

Pyroptosis is a programmed cell death characterized by the breakdown of cell membranes and the release of cell contents, usually accompanied by the activation of inflammatory responses (8). NLRP3 inflammasome is an important intracellular immune sensing complex, which participates in the activation of the typical pyroptosis pathway and plays an important role in the occurrence and development of a variety of diseases (9, 10). The NLRP3 inflammasome consists of NLRP3, ASC, and Caspase-1 precursors. Among them, NLRP3 is a three-part protein containing PYD, NACHT and LRR domains (11). As a sensor, NLRP3 inflammasome can recognize a variety of endogenous and exogenous stimuli, such as pathogens, cell damage, and metabolites (12). NLRP3 inflammasome activation is usually divided into two stages: initiation and activation (13). During the initiation phase, various pathogen-associated molecular patterns (PAMPs) and damage-associated molecular patterns (DAMPs) are recognized by their corresponding receptors that trigger nuclear factor-κB (NF-κB) nuclear translocation, leading to the transcriptional activation of NLRP3, pro-IL-1β, and pro-IL-18. During the activation phase, NLRP3 undergoes conformational changes due to changes in the intra- and extracellular environments (e.g., intracellular mitochondrial dysfunction and reactive oxygen species (ROS) production, calcium inflow and potassium outflow, and lysosomal damage; extracellular ATP, perforin, RNA viruses, particulate matter, etc.), binds to the precursor of Caspase-1 through the bridging ASC to form an aggregation and ultimately activates Caspase-1, leading to the cleavage of gasdermin D (GSDMD) and release of its GSDMD-N structural domain to penetrate the cell membrane to release inflammatory factors such as IL-1β and IL-18 (5, 14). The study of this process provides a theoretical basis for the development of NLRP3 inhibitors, which may provide a new strategy for the treatment of related inflammatory diseases (**Figure 1**).

### 2.2 Role of NLRP3 in SCIRI

The role of NLRP3 and pyroptosis in the spinal cord has attracted increasing attention. Xu et al. conducted RT-PCR quantitative analysis of peripheral blood of patients with SCI and found that the expression of NLRP3 or GSDMD was positively correlated with the degree of SCI (15). Wang et al. performed ELISA and RT-qPCR on spinal cord tissue samples from mice 7 days after SCI, and the results showed that the mRNA levels of IL-18, IL-1β, Toll-like receptor 4 (TLR4) and NLRP3 were significantly increased. Subsequent *in vitro* and *in vivo* experiments showed that TLR4 promoted microglial pyroptosis through JAK2/STAT1/DDX3X/NLRP3 axis to aggravates SCI (16). SCIRI, a specific SCI, can increase the phosphorylation of NF-κB p65 in the spinal cord, the activation of caspase-1 mediated by NLRP3 inflammasome, and subsequent inflammatory factors to induce pyroptosis (6). In the pathological process of SCIRI, the intracellular ATP level decreases, leading to mitochondrial dysfunction, and then produces a large number of ROS. These ROS can damage cell membrane, protein and DNA, and eventually lead to apoptosis or necroptosis (1, 2). In addition, damaged nerve cells and glial cells will release a variety of pro-inflammatory factors, such as tumor necrosis factor (TNF) and interleukin (IL), which attract immune cells to infiltrate and aggravate nerve injury (17). The apoptosis effectors BAX and BAK can induce the activation of the NLRP3 inflammasome and the secretion of IL - 1β by inducing mitochondrial outer membrane permeability (MOMP) and releasing pro - apoptotic factors, which in turn activate caspases - 3 and - 7. The necroptosis effectors RIPK3 and MLKL can activate the NLRP3 inflammasome by promoting ROS production and K^+^ efflux. GPX4, an important negative regulator of ferroptosis, may also be involved in inhibiting the activation of the NLRP3 inflammasome (18). Although the inextricable link between NLRP3 inflammasome activation and cell death has not been reported in SCIRI studies, there is some circumstantial evidence. Qiu et al. found that after cerebral ischemia/reperfusion injury in rats, the increased level of Vav1 (a guanine nucleotide exchange factor related to microglia activation) promotes the activation of microglia and NLRP3 inflammasome to increase inflammation and neuronal apoptosis in rats (19). Western blot results showed that not only the expression of pyroptosis-related proteins such as NLRP3 and cleaved caspase-1 was increased in the retinal neurons with ischemia/reperfusion injury *in vivo* and *in vitro*, but also the expression of NLRP3 and cleaved caspase-1 was increased. Moreover, cleaved caspase-3, BAX, RIPK3 and MLKL were also significantly increased, indicating that NLRP3 mediated pyroptosis occurred during ischemia/reperfusion, accompanied by apoptosis and necroptosis (20). Therefore, the regulation of NLRP3 inflammasome may become a new target for SCIRI intervention, and related inhibitors have shown good protective effects in animal experiments, suggesting the prospect of clinical application (2, 21).

## 3 METTL3-mediated regulation of NLRP3 and pyroptosis

METTL3 is the core catalytic component of the m6A methyltransferase complex and has become a key epigenetic regulator of inflammatory responses. Emerging evidence suggests that m6A-mediated RNA modification links METTL3 to activation of the NLRP3 inflammasome. In the study of endothelial cell injury, METTL3 catalyzes the m6A modification of NLRP3 mRNA, and then promotes the expression of NLRP3 protein. This process depends on the involvement of m6A reading protein YTHDF1, and finally regulates the pyroptosis process of endothelial cells and affects the inflammatory response of soft tissue injury (22). Up-regulation of METTL3 increases the expression level of MALAT1 through m6A methylation modification to promote the degradation of USP8 mRNA, thereby regulating the ubiquitination and protein stability of TAK1, and promoting macrophage pyroptosis and inflammation (23). Zhou et al. found that human umbilical cord mesenchymal stem cell-derived extracellular vesicles interacted with METTL3 to reduce the m6A level of NLRP3 mRNA in macrophages to inhibit the secretion of pro-inflammatory factors and alleviate knee osteoarthritis in mice (24). Zhang et al. concluded that METTL3 is up-regulated in liver I/R injury and regulates TXNIP expression through m6A modification, which promotes NLRP3 inflammasome assembly and caspase-1-dependent pyroptosis, thus playing a role in hepatocyte injury (25).

Although direct studies in SCIRI are limited, similarities exist in the associated neuropathology. In cerebral ischemic stroke, METTL3 stabilizes NLRP3 mRNA through m6A modification and amplifies pyroptosis and inflammation (26). In microglia of acute brain injury (TBI) patient/mouse model, m6A levels and METTL3 protein expression were significantly upregulated, which then promoted BATF protein expression by stabilizing BATF mRNA. As a transcription factor, BATF directly binds to a variety of inflammatory cytokine and chemokine genes to drive inflammatory responses. In contrast, knockdown of METTL3 or pharmacological inhibition of METTL3 reduced the release of pro-inflammatory cytokines (such as IL-6, IL-12, and TNF-α), reduced neutrophil infiltration, and improved neuronal survival and neurological function (27). This highlights its dual role as both a driver and a potential therapeutic target in neuroinflammation.

## 4 Therapeutic Strategies Targeting the METTL3-NLRP3 Axis

Inhibition of the METTL3-NLRP3 axis is a promising therapeutic avenue to attenuate pyroptosis and neuroinflammation in SCIRI. Although not yet tested in SCIRI, therapeutic effects have been demonstrated in different animal and cellular models. Strategies against METTL3 mainly focus on inhibiting its activity, and selective METTL3 inhibitors reduce the overall m6A level by competitively binding to the S-adenosylmethionine (SAM) binding pocket of METTL3. F039-0002 and 7460-0250 bind tightly to METTL3 protein, blocking its catalytic pocket and increasing YTHDF3-mediated phosphoglycerate phosphatase (PGP) expression. This reprograms macrophage glucose metabolism and inhibits CD4+ T helper 1 (Th1) cell differentiation, thereby effectively alleviating dextran sodium sulfate-induced colitis (28). In a rat SCI model, STM2457 induces ATG5-mediated autophagy by inhibiting METTL3-mediated m6A modification of miR-30c, thereby alleviating secondary neuronal injury and promoting functional recovery (29). Similarly, Coptisine chloride, a natural alkaloid extracted from Coptis chinensis, competitively binds to the SAM binding site to inhibit its methyltransferase activity. This affects the stability of TNFAIP3 and ubiquitination of NEK7, ultimately inhibiting NLRP3 inflammasome activation and pyroptosis, and improving the therapeutic effect of periodontitis (30). In addition, knockdown of METTL3 by tail vein injection of lentivirus carrying METTL3 siRNA in rats reduced cardiac fibrosis and improved cardiac function by inhibiting cardiac fibroblast activation and collagen synthesis (31).

Strategies targeting the NLRP3 inflammasome aim to disrupt its assembly, thereby inhibiting inflammation. MCC950 specifically inhibits NLRP3 activation and ASC oligomerization by binding to the Walker B site of the NLRP3 NACHT domain, thereby inhibiting its ATP hydrolysis and the closure of its active conformation (32). In a C57Bl/6 mouse model of ischemic stroke, relative NLRP3 gene expression levels increased 20- to 30-fold within 1 day in the ischemic hemisphere. However, treatment with MCC950 significantly reduced infarct volume, caspase 1 activation, immune cell infiltration in the ischemic hemisphere, and preserved blood-brain barrier integrity (33). Other inhibitors such as CY-09 and C77 can also exert inhibitory effects by binding to the NACHT domain of NLRP3 (11). Additionally, studies have found that natural compounds with antioxidant properties such as curcumin, resveratrol, quercetin, ginsenosides, and mangiferin can effectively inhibit NLRP3 inflammasome activation by suppressing key signaling molecules including NF-κB, COX-2, and iNOS, thereby reducing production of pro-inflammatory cytokines IL-1β and TNF-α (34). Furthermore, combination with other therapeutic modalities such as electroacupuncture and drugs may enhance the inhibitory effects on NLRP3 inflammasome (35).

## 5 Conclusion and perspectives

Through this review, we provide an in-depth exploration of the critical role of NLRP3 inflammasome in pyroptosis and the mechanisms by which NLRP3 inflammasome regulation impacts neural cells. METTL3, which plays a crucial role in RNA methylation, demonstrates unique biological functions in inflammatory response modulation. Current studies indicate that the role of METTL3 extends beyond NLRP3 inflammasome regulation and may involve other inflammation-related pathways and cell death processes. Furthermore, future SCIRI (Spinal Cord Ischemia-Reperfusion Injury) research should also focus on the interactions between METTL3 and other signaling pathways to gain a comprehensive understanding of its biological functions.

Despite the progress made in current studies, there remain critical obstacles. METTL3 regulates global RNA methylation, and systemic inhibition may disrupt homeostasis. Future studies are also needed to delve into the mechanism of METTL3 in different disease models and evaluate its safety and efficacy as a therapeutic target (36). Cell type-specific delivery systems (e.g., nanoparticles targeting microglia) are needed to minimize off-target effects (37). Existing research has mainly centered on the acute phase. There is a need for chronic SCIRI models to assess the long - term therapeutic effects and the potential for nerve regeneration. Moreover, it remains to be determined whether the targeted suppression of both METTL3 and NLRP3 will bring about additional benefits. Although some headway has been made in understanding the METTL3 - NLRP3 axis, there is a scarcity of research on SCIRI. Direct evidence is still required to fill this knowledge void.

Overall, exploring the interactions between METTL3 and NLRP3, as well as related molecules, not only helps to elucidate the pathogenesis of SCIRI but may also provide potential therapeutic targets for clinical therapy.

## Glossary

SCIRI: spinal cord ischemia-reperfusion injury
NLRP3: nucleotide-binding oligomerized structural domain-like receptor protein 3
SCI: spinal cord injury
METTL3: methyltransferase-like 3
m6A: N6-Methyladenosine
ROS: reactive oxygen species
TNF: tumor necrosis factor IL, interleukin
GSDMD: Gasdermin D
PAMPs: pathogen-associated molecular patterns
DAMPs: damage-associated molecular patterns
JOA: Japanese Orthopedic Association
NDI: neck disability index
TLR4: toll-like receptor 4
